# The Association Between COVID-19, Air Pollution, and Climate Change

**DOI:** 10.3389/fpubh.2021.662499

**Published:** 2021-07-06

**Authors:** Teerachai Amnuaylojaroen, Nichapa Parasin

**Affiliations:** ^1^School of Energy and Environment, University of Phayao, Phayao, Thailand; ^2^Atmospheric Pollution and Climate Change Research Unit, School of Energy and Environment, University of Phayao, Phayao, Thailand; ^3^School of Allied Health Science, University of Phayao, Phayao, Thailand

**Keywords:** COVID-19, air pollution, climate change, environment, environmental impact

## Abstract

This mini-review aims to highlight both the positive and negative relationship between COVID-19 and air pollution and climate change based on current studies. Since, COVID-19 opened a bibliographic door to scientific production, so there was a limit to research at the moment. There were two sides to the relationship between COVID-19 and both air pollution and climate change. The associated with climate change, in particular, defines the relationship very loosely. Many studies have revealed a positive correlation between COVID-19 and each air pollutants, while some studies shown a negative correlation. There were a few studies that focused on the relationship between COVID-19 in terms of climate. Meanwhile, there were many studies explained the relationship with meteorological factors instead.

## Introduction

COVID-19, an infectious respiratory infection, rapidly spread to other parts of China and eventually became a global epidemic (1–3). Several early studies shown that being in close proximity to anyone who has the disease increases the risk of infection (4–6). Furthermore, recent review papers state that there are two modes of COVID-19 transmission: direct and indirect. SARS-CoV-2 bioaerosol is transferred in direct mode through the air in the internal atmosphere in the form of droplet nuclei and other body fluids, as well as from mother to infant. In the indirect mode, infections are caused by fomites or the surfaces of furniture and fixtures in the immediate environment of an infected patient (7, 8). According to recent research, air pollutants are risk factors for infectious respiratory disease as well as COVID-19 (9–12). Air pollution can make patients more vulnerable to the disease and have a detrimental effect on their prognosis (13–15). This could be explained by enhanced angiotensin converting enzyme 2 (ACE-2) expression in respiratory epithelial cells exposed to air pollution (16). COVID-19 lockdown, on the other hand, has had numerous positive effects on the environment and energy consumption (17). It was loosely defined in terms of climate change and COVID-19. However, many of the underlying causes of climate change are also linked to an increase in pandemics through climate factors such as temperature, wind speed, and humidity (18–20). Recent studies have indicated a mostly negative relationship between COVID-19, air pollution, and climate change, for example, the relationship between climate change, environmental pollution, COVID-19, and mental health (21), and the influence of air pollution on COVID-19 mortality (22). However, further research is needed to determine if it is relevant or not. The highlight of this mini-review is the state of both the relationship between COVID-19 and air pollution and climate change, as well as revealing both positive and negative effects of the relationship.

## The Relationship Between COVID-19, Air Pollution and Climate Change

### Covid-19 and Air Pollution

**Figure 1:**
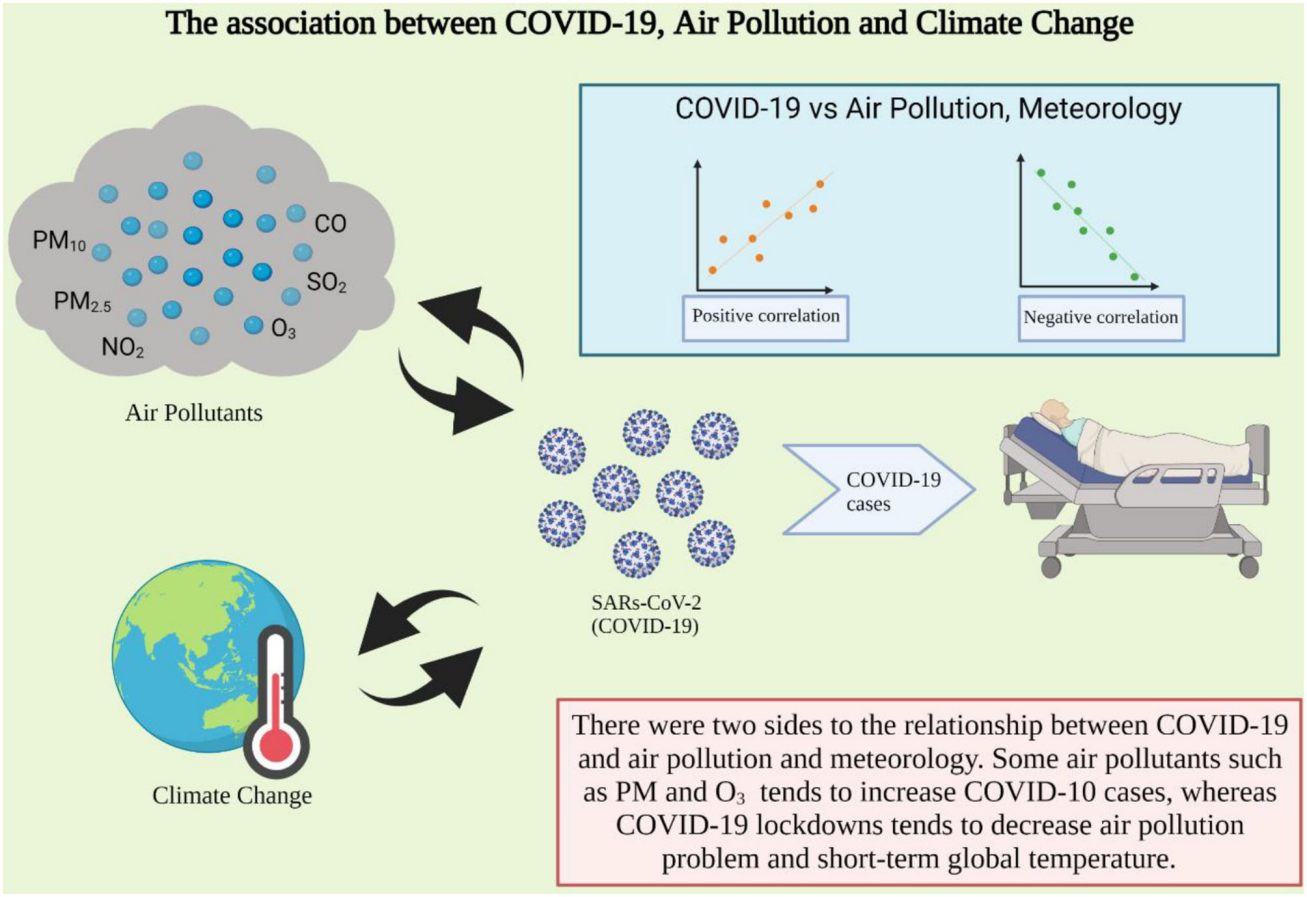
The association between COVID-19 and air pollution and climate change (Created with BioRender.com).

The relationship between air pollution and coronavirus is ambiguous, which was indicated by both positive and negative correlation in the current studies (Figure 1). Many studies have revealed that air pollution increases the COVID-19 mortality rate, whereas COVID-19 lockdown could reduce air pollution level at the same time. There is some evidence from recent studies revealing that increased risk of COVID-19 infection is associated with exposure to higher air pollutant levels including PM_2.5_, PM_10_, CO, NO_2_, SO_2_, and O_3_. For example, Wu et al. (23) and Cole et al. (24), have found that long-term exposure to PM_2.5_ was associated with a substantial 11% increased risk of cardiovascular death and hospital admissions. In addition, according to a study conducted in Milan, Italy, all recorded daily new COVID-19 cases were positively correlated with the average surface PM_2.5_ and the daily maximum PM_10_ (25). Several studies discussed the relationship between O_3_ and NO_x_ and COVID-19 but it was unclear in those studies. The current research discovered both a negative (26) and a positive (25) correlation between NO_x_ and COVID-19 in various parts of the world. However, as the role in flagging SARS-CoV-2 transmission, O_3_ was importantly correlated with COVID-19 (27). Unfortunately, there has been relatively little research into the relationship between CO and SO_2_. Sangkham et al. (26) discovered that increases in CO and SO_2_ were correlated with decreases in the daily number of COVID-19 cases, while another study recently indicated that 1 g/m^3^ CO is associated with a 15.11% increase in the daily count of confirmed COVID-19 cases (28). Meanwhile, a SO_2_ concentration of 10 g/m^3^ was associated with a 7.79% decrease in the regular number of reported cases in China's 120 cities (28). As a result, there is insufficient evidence for conclusive findings for CO and SO_2_ in the current study, leading to these variables being both negatively and positively associated with SARS-CoV-2 transmission. At the same time, some studies indicated that COVID-19's indirect effect could help alleviate the problem of air pollution. For example, Mostafa et al. (29) reported a strong correlation between COVID-19 lockdown and a 15–33% reduction in NO_2_ and 5% reduction throughout CO in Egypt. Kumari and Toshniwal (30), studied a similar situation that occurred in India. They estimated that the COVID-19 lockdown could reduce PM_10_, PM_2.5_, NO_2_, and SO_2_ concentrations by 55, 49, 60, and 19%, respectively, in Delhi and Mumbai. This finding was corroborated by Wetchayont (31), who discovered that several air pollutant concentrations, including PM_2.5_, PM_10_, O_3_, and CO, showed a significant decline during the COVID-19 outbreak in Bangkok, Thailand.

### Covid-19 and Climate Change

When compared to air pollution, the link between COVID-19 and climate change is extremely ambiguous. Since COVID-19 events were detected in 2019, there was no clear evidence of climate change's effect on COVID-19, which is typically a long-term event (32). There was a recent review about climate change and the COVID-19 epidemic in Rodó et al. (33). They stated that both absolute humidity (AH) and temperature have been linked to influenza outbreaks and facilitated epidemic progression. In tropical regions, SARS-CoV-2 appears to have a higher airborne survival and transmission rate than the influenza virus. A blockade of aerosol transmission in the case of influenza virus has been described for temperature variations above 30°C. Whereas, the direct effect of the pandemic-driven response will be negligible, with a cooling a global temperature of around 0.01 ± 0.005°C by 2030 (34). This result was supported by the concept of anthropause that suggested that reduced human mobility during the pandemic will reveal critical aspects of our impact on animals, providing important guidance on how best to share space on this crowded planet (35). The global temperature signal, however, is anticipated to be small as a result of the pandemic's short-term dynamics. Another evidence that might correlate the relationship between climate and COVID-19 is using meteorological factors including the temperature, Relative Humidity (RH), AH, and Wind Speed (WS). Most studies in many countries, including Thailand, Singapore, India, and China, found a positive relationship between temperature and the daily number of COVID-19 cases (26, 36, 37). Several research, however, found a negative association between temperature and COVID-19 cases. Wu et al. (38) and To et al. (39), for example, discovered that rising temperatures were associated with a decrease in daily new cases of COVID-19 in Brazil and Canada. Other variables, such as RH (%), AH (g/m^3^), and WS (m/s), had a positive relationship with the number of COVID-19 cases in many countries, including Thailand and Turkey (26, 40). Furthermore, relative humidity was found to be negatively correlated with daily new COVID-19 cases by Wu et al. (38). Another research provided experimental results demonstrating that lower relative humidity promotes influenza virus transmission, confirming this weather effect (41). The SARS coronavirus's stability at low humidity can support its spread in communities in subtropical climates (such as Hong Kong) during the spring (42). For WS, the relationship with COVID-19 was identical to those of humidity and temperature, which have both positive (26) and negative (43) relationships. WS, on the other hand, may potentially affect SARS-CoV-2 in an indoor rather than outdoor environment among symptomatic and asymptotic cases due to sneezing and coughing in population-dense areas.

## Conclusion

The conclusion is based on the current studies on the relationship between COVID-19 and air pollution and climate change. Many studies have shown that several air contaminants, including PM_2.5_, PM_10_, CO, NO_2_, SO_2_, and O_3_, increase COVID-19 mortality rates. However, some research suggests that COVID-19's indirect effect could help to reduce air pollution. Particulate matter has been linked to a rise in COVID-19 incidents. Although the current study lacks adequate evidence for conclusive findings for CO and SO_2_, which are both negatively and positively associated with SARS-CoV-2 transmission. Current research has discovered both a negative and a positive relationship between NO_x_ and COVID-19 in different parts of the world. As a marker of SARS-CoV-2, O_3_ was found to be significantly associated with COVID-19 and air pollution-related infections. While there has been no conclusive evidence of the effect of climate change on COVID-19, which is typically a long-term phenomenon. A few studied suggested that SARS-CoV-2 has a higher airborne survival and transmission rate than the influenza virus in tropical areas. While the direct effect of the pandemic-driven response will reduce a global temperature by 2030. The relationship between temperature, humidity, and wind speed can be used to support the correlation between the atmosphere and COVID-19 events. Since there are uncertainty in the relationships between COVID-19 and air pollution and climate change, the meta-analysis should be conducted in the future work.

## Author Contributions

TA: conceived and designed the manuscript, contributed reagents and materials, and wrote the paper. NP: editing and revised the paper. All authors contributed to the article and approved the submitted version.

## Conflict of Interest

The authors declare that the research was conducted in the absence of any commercial or financial relationships that could be construed as a potential conflict of interest.
